# Enhanced Lipid Production and Molecular Dynamics under Salinity Stress in Green Microalga *Chlamydomonas reinhardtii* (137C)

**DOI:** 10.3390/md17080484

**Published:** 2019-08-20

**Authors:** Thanapa Atikij, Yolani Syaputri, Hitoshi Iwahashi, Thanit Praneenararat, Sophon Sirisattha, Hakuto Kageyama, Rungaroon Waditee-Sirisattha

**Affiliations:** 1 Department of Microbiology, Faculty of Science, Chulalongkorn University, Phayathai Rd., Pathumwan, Bangkok 10330, Thailand; 2 The Chemical Approaches for Food Applications Research Group, Faculty of Science, Chulalongkorn University, Phayathai Rd., Pathumwan, Bangkok 10330, Thailand; 3 Graduate School of Applied Biological Sciences, Gifu University, 1-1 Yanagido, Gifu, 501-1193, Japan; 4 Department of Chemistry, Faculty of Science, Chulalongkorn University, Phayathai Rd., Pathumwan, Bangkok 10330, Thailand; 5 Thailand Institute of Scientific and Technological Research (TISTR), Khlong Luang, Pathum Thani 12120, Thailand; 6 Graduate School of Environmental and Human Sciences, Meijo University, Nagoya 468-8502, Japan

**Keywords:** salt stress, lipid production, microalgae, malonyl-ACP, salinity

## Abstract

Microalgal lipids are a source of valuable nutritional ingredients in biotechnological industries, and are precursors to biodiesel production. Here, the effects of salt-induced stresses, including NaCl, KCl, and LiCl stresses, on the production of lipid in green microalga *Chlamydomonas reinhardtii* (137c) were investigated. NaCl stress dramatically increased saturated fatty acids (SFAs), which accounted for 70.2% of the fatty acid methyl ester (FAMEs) under stress. In contrary, KCl stress led to a slight increase in SFAs (47.05%) with the remaining being polyunsaturated fatty acids (PUFAs) (45.77%). RT-PCR analysis revealed that the genes involved in FA biosynthesis, such as *PDH2*, *ACCase*, *MAT* and *KAS2*, were up-regulated by NaCl-induced stress. Conversely, the genes responsible for the Kennedy pathway were suppressed. The alteration of FA homeostasis was further assessed by overexpressing MAT, the enzyme responsible for the production of malonyl-ACP, a key building block for FA biosynthesis, in the cyanobacterium *Synechococcus elongatus* PCC 7942. Intracellular FA composition was affected, with a predominant synthesis of SFAs in transformed cells. Owing to the diversity and relative abundance of SFAs, monounsaturated fatty acid (MUFAs) and PUFAs enable the feasibility of using microorganisms as a source of microalgal lipids or valuable nutritional ingredients; salt-induced stress and expression of MAT are useful in providing precursors for enhanced lipid production.

## 1. Introduction

Microalgae are a group of plant-like, unicellular photosynthetic organisms with ubiquitous distribution. Given the expansive diversity of microalgae in nature, a diverse assortment of fatty acids (FAs) and lipid classes of nutritional importance are biosynthesized in microalgae. FA and lipid compositions vary substantially among phylogenetic groups [1,2]. For instance, green microalgae predominantly accumulate SFAs and MUFAs, together with trace amounts of PUFAs. Contrastingly, chromalveolate microalgae such as diatoms contain significant amounts of PUFAs. Comprehensive studies on FA profiles and their distribution patterns in microalgae have been summarized in a previous review article [1].

Recently, microalgal cells were suggested as veritable biochemical factories. They have higher growth rates and higher photosynthetic efficiencies than terrestrial plants because of efficient CO_2_ fixing capability. Additional advantages include the use of non-arable land, minimal and basic nutritional requirements, and adaptation to a wide range of environmental conditions [3]. Algal lipid accumulation concomitantly occurs with alteration of the lipid biosynthetic pathways for storage as triacylglycerols (TAGs), and most microalgae contain intracellular lipid bodies. TAGs are used as feedstock for direct conversion to biodiesel. Some microalgal species, such as *Chlorella vulgaris* [4], *Nanochloropsis* sp. [5], and *Botryococcus braunii* [5,6] are known as natural lipid accumulators because of a very high lipid content, ranging between 55–75% of intracellular storage lipids.

Lipid biosynthesis in microalgae initiates the chloroplast by CO_2_ fixation and subsequent conversion into pyruvate via the Calvin cycle and Glycolysis pathway, respectively [7]. Acetyl CoA derived from pyruvate is subsequently utilized to initiate FA biosynthesis using various enzymes, such as the pyruvate dehydrogenase complex containing pyruvate dehydrogenase (PDH), acetyl-coenzyme A carboxylase (ACCase), acetyl-CoA:acyl carrier protein (ACP) transacylase, and malonyl CoA-acyl carrier protein transacylase (MAT). The condensation of acetyl-CoA and malonyl-CoA results from an enzymatic reaction catalyzed by MAT and subsequently mediated by 3-ketoacyl-ACP synthase (KAS) to form 3-ketoacyl-ACP, which is a substrate for FA elongation. After each condensation, a reduction, dehydration, and second reduction occur, and the next steps are catalyzed by enzymes known collectively as the FAS complex [8]. Next, fatty acyl-ACP thioesterase (FAT) produces free FA chains by hydrolysis of fatty acyl-ACPs produced by the FA biosynthetic pathway [9], and free FAs are subsequently released out of the chloroplast into the cytosol and used as a precursor for TAG biosynthesis. Among de novo pathways for TAG biosynthesis, the Kennedy pathway is recognized as the main pathway in microalgae [10].

There has been a wide range of studies performed to identify and enhance lipid accumulation in microalgae, such as nutrient depletion [11], high light stress [12], salt stress [13], and increased carbon dioxide concentration [14]. Lipids typically provide a storage function in the cell that enables microalgae to endure adverse environmental conditions. In addition, metabolic engineering is a promising strategy to improve lipid production in microalgae. Approaches for genetic engineering based on the target of interest include enhancement of FA or TAG biosynthesis, regulation of the PDH-bypass pathway, inhibition of the competitive pathway, and transcriptional engineering [15].

Here, we employed the green microalga Chlamydomonas as a model to study the dynamics of lipid metabolism. Effects of salt stresses, including NaCl, KCl, and LiCl on intracellular lipid accumulation were investigated. These salts caused dramatic changes on cell morphology and lipid accumulation through distinct mechanisms. Expression analysis of genes involved in FA and TAG biosynthesis pathways revealed that FA biosynthesis was up-regulated, but TAG biosynthesis was down-regulated by salt stress. Further, overexpression of the *mat* gene modulated the pattern of FA production in expressing cells of the cyanobacterium *Synechococcus elongatus* PCC 7942. The dynamic changes of lipid metabolism, together with molecular analyses, presented in this study provide insights into the mechanisms of salt-induced stress. Because the diversity and relative abundance of FAs are key properties in the feasibility of using microalgae as a source of lipids or valuable nutritional ingredients, salt-induced stress and expression of MAT are useful in providing precursors for lipid production.

## 2. Results and Discussion

### 2.1. Salts Trigger Intracellular Lipid Accumulation in C. reinhardtii (137c)

**Figure 1:**
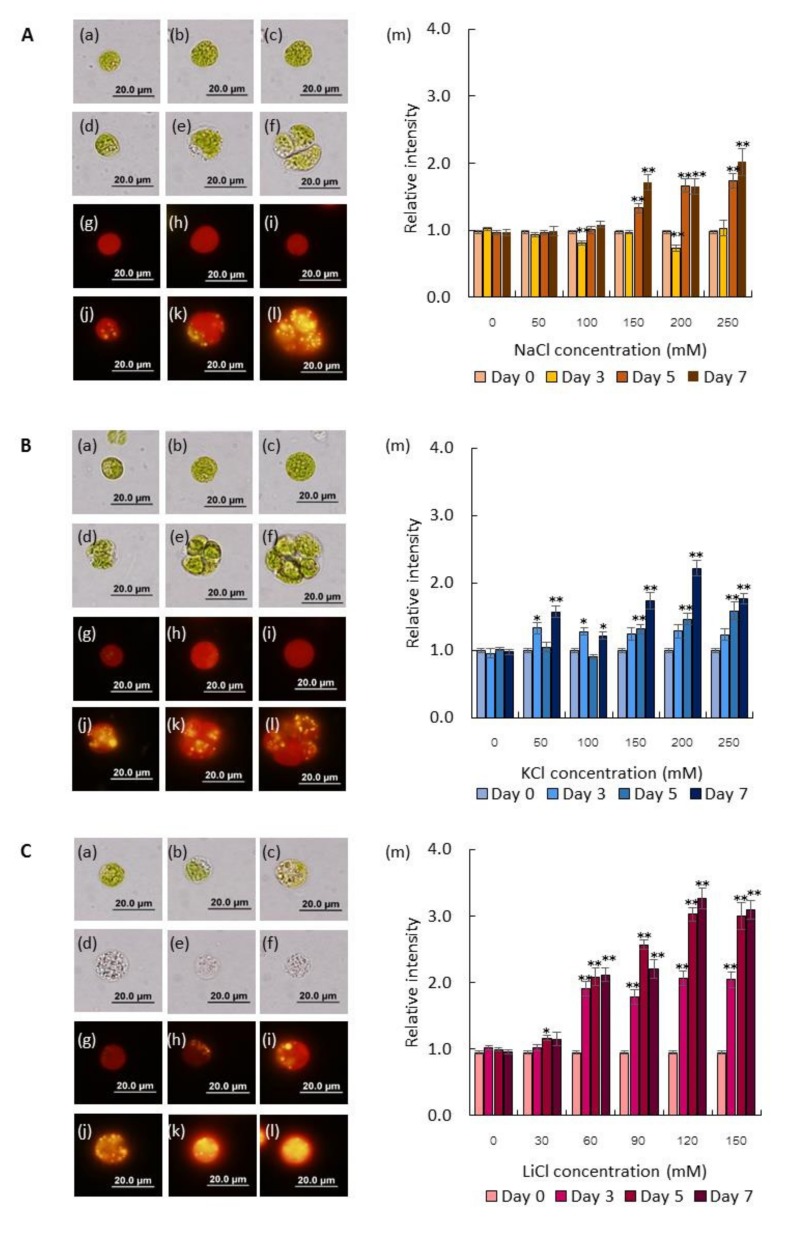
Effects of salt stress on morphology and lipid accumulation. (**A**) NaCl stress. (**B**) KCl stress. (**C**) LiCl stress. *C. reinhardtii* (137c) cells observed under a light microscope (a-f) and fluorescent microscope (g-l). Cells at the exponential growth phase were subjected to salt stress (NaCl or KCl) at various concentrations, including 0 mM (a, g), 50 mM (b, h), 100 mM (c, i), 150 mM (d, j), 200 mM (e, k), and 250 mM (f, l) for 7 days. In the case of LiCl, the concentrations were 0 mM (a, g), 30 mM (b, h), 60 mM (c, i), 90 mM (d, j), 120 mM (e, k), and 150 mM (f, l) for 7 days. Morphology and lipid droplets were observed by Nile red staining, as described in Materials and Methods. Relative intensity of lipid content under NaCl stress was analyzed using ImageJ software (M). Data are expressed as mean ± SE. * *p* < 0.01, ** *p* < 0.001, unpaired T-test (*n* ≥ 10).

The effects of salts (NaCl, KCl, and LiCl) on intracellular lipid droplet accumulation were investigated. Cells were subjected to salts at indicated concentrations for 0, 3, 5, and 7 days. Staining for the detection of intracellular lipid was conducted using Nile red staining. This fluorescent dye is one of the most popular stains used to assess lipid content, especially neutral lipids produced by microalgae. Dynamic changes of lipid accumulation and cell morphology are shown in Figure 1A–C. In the case of NaCl stress, yellow spots were first observed on day 3 with a concentration of 250 mM (Figure 1A). Lipid droplet contents increased with increasing NaCl concentrations. The highest lipid accumulation was observed on day 7 with a concentration of 250 mM (2.01 ± 0.20 fold). Cell size upon NaCl stress was slightly larger than that of the control cells. After treatment with high NaCl concentration (i.e., 250 mM) for seven days, algal cells were found to have a palmelloid morphology rather than a unicellular morphology. KCl stress led to morphological alteration and lipid accumulation, with trends similar to that of NaCl stress (Figure 1B). However, the highest lipid accumulation was observed earlier than that of the NaCl stress. Subjecting microalgae to a concentration of 200 mM KCl for 5 days led to a 2.22 ± 0.11 fold increase in lipid accumulation. Similar to NaCl, pamelloid formation was pronounced under KCl stress.

LiCl was used as an additional salt stressor. In this case, lipid accumulation was tested at lower concentration ranges due to LiCl toxicity [16]. Unlike NaCl and KCl, LiCl did not cause pamelloid formation in any conditions tested (Figure 1C). It should be noted that LiCl robustly affected cell viability. Cell bleaching was visible after treatment for three days. However, lipid droplet signals were higher than both NaCl and KCl. The highest signal was 3.27 ± 0.16 fold on day 7 under 120 mM LiCl. Details of the cell morphology and lipid body accumulation dynamics over seven days of stress with each salt are shown in Supplementary Materials Figures S1–S3. Additionally, we assessed the effects of sodium acetate on microalgal lipid accumulation, as sodium acetate is a well-known substrate for acetyl-CoA-production. As shown in Figure S4, lipid droplets increased with increasing sodium acetate concentration. The highest lipid content was found on day 3 with a concentration of 250 mM (2.04 ± 0.09 fold). Thereafter, lipid droplet signals gradually declined. 

The palmelloid morphology is a multicellular stage of unicellular algal cells, and is particularly robust in Chlamydomonas [17]. Extreme environmental stressors or even predators could trigger palmelloid formation [18,19]. Our results demonstrated that NaCl and KCl, but not LiCl or sodium acetate, triggered palmelloid formation (Figure 1), suggesting that NaCl and KCl influenced the expression of gene(s) involved in palmelloid formations or polysaccharide metabolism, such as biofilm production. In *E. coli*, expression of *bolA*, the gene associated with biofilm formation, was up-regulated in response to several forms of stress, including carbon starvation, osmotic stress, heat shock, acidic stress, and oxidative stress [20,21]. Transformation of *E. coli* cells with the *Chlamydomonas bolA* -like gene, an ortholog of *E. coli bolA*, altered cell morphology and increased biofilm formation [22]. The molecular mechanism of palmelloid formation in reaction to specific salt stresses is an interesting topic for future investigation. 

### 2.2. Dynamics of Intracellular Fatty Acids during Salt Stress

To gain insight into intracellular FA composition and quantification during salt stresses, FAMEs analysis was performed. It should be noted that 250 mM NaCl resulted in the highest lipid accumulation observed by Nile red staining (Figure 1A). Nonetheless, this condition caused cell death within 3–5 days of cultivation. Thus, NaCl concentration was reduced to 200 mM for stress treatment and FAMEs analyses. Table S1 summarizes the amount of intracellular FAs in *C. reinhardtii* (137c) under control (non-stress) and salt stress conditions (by 200 mM NaCl, 200 mM KCl, and 120 mM LiCl). Under control conditions, total SFAs were 38.79 ± 0.33% of total FA, while total MUFAs were very low (5.91 ± 0.66% of total FA). Total PUFAs comprised of 56.58 ± 1.03% of total FA. Linoleic acid (C18:2) was determined to be the major component of FA in this microalga, as this was the highest content of total FAs (33.64 ± 0.04%), followed by palmitic acid (C16:0). In *Chlamydomonas* sp., palmitic acid (C16:0) is a major FA component [23,24]. In our study, we found that the FA profile was altered by salt stress. Total SFAs significantly increased to 50.48 ± 6.67% of total FA within three days of cultivation. Total SFA was highest on day 7, and accounted for 70.19 ± 0.00% of total FA (Table S1). Total MUFA content remained unchanged. Contrastingly, total PUFAs dramatically declined as cultivation time increased. PUFAs were reduced to the lowest level at 23.06 ± 0.00% of total FA on day 7.

**Figure 2:**
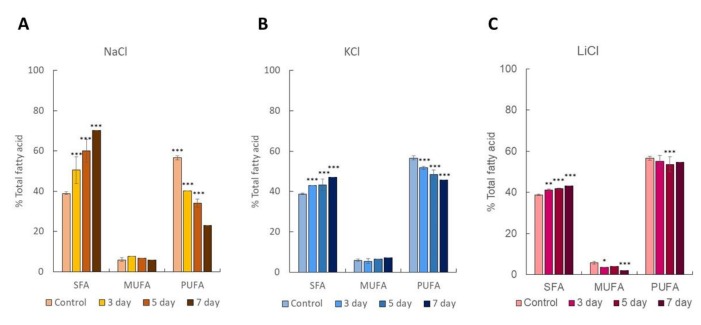
SFA, MUFA and PUFA contents of salt-stressed cells, including (**A**) 200 mM NaCl, (**B**) 200 mM KCl, and (**C**) 120 mM LiCl. FAMEs were analyzed as described in Materials and Methods. SFA; saturated fatty acid, MUFA; mono-unsaturated fatty acid, PUFA; poly-unsaturated fatty acid. *p < 0.001, two-way ANOVA (*n* = 3).

In the case of KCl or LiCl, total SFAs significantly increased compared to control conditions. Total SFAs reached the maximum level after seven days of cultivation. SFAs accounted for 47.05 ± 0.00% and 43.09 ± 0.00% of total FA under KCl and LiCl stresses, respectively. All of the salt stresses decreased PUFAs as in NaCl stress, but to a lesser extent. Our results suggested that simple stress by salts (NaCl, KCl, LiCl) enhanced SFA composition. The diversity and relative abundance of SFAs, MUFAs, and PUFAs are key properties in determining the feasibility of using a microorganism as a source for algal lipid, biofuel feedstock, biomaterial synthesis or high-value nutrient supplements. In biodiesel production, medium chain fatty acids (MCFAs) are preferable. In this study, we compared the changes of MCFAs under control and salt stress. Myristic acid (C14:0), palmitic acid (C16:0) and palmitoleic acid (C16:1) were all increased on seven days of exposure to NaCl stress (Figure 2 and Table S1). Among these FAs, palmitic acid was drastically increased (1.87 fold). Long chain fatty acids, including stearic acid (C18:0) and arachidonic acid (C20:0) slightly increased, while the content of oleic acid (C18:1) was unchanged. These results implied that NaCl mainly increased MCFA content, especially palmitic acid. In *Dunaliella* sp., NaCl was a strong inducer for lipid accumulation [25]. MUFA content was dramatically increased (5.1% to 26.93% of total FA), followed by SFA content (33.62% to 46.27% of total FA). Conversely, total PUFAs were decreased. Nevertheless, the mechanisms for salt stressed-induced changes in lipid composition remain elusive. Therefore, we further examined the transcriptional level of genes involved in lipid biosynthesis.

### 2.3. Gene Expression Analysis

**Figure 3:**
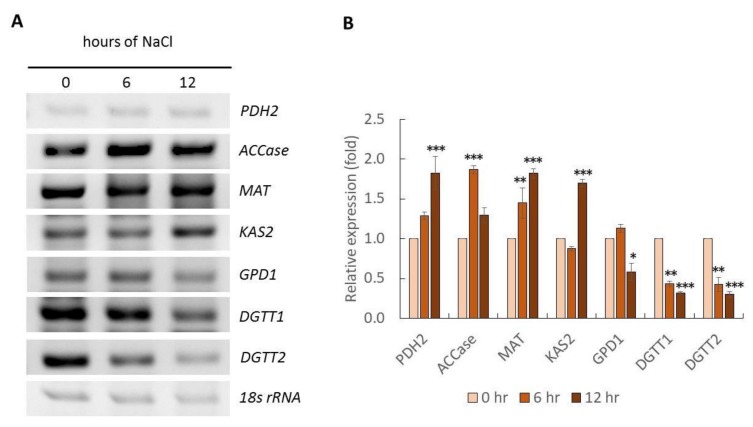
Semi quantitative RT-PCR analysis of stressed cells treated with 200 mM NaCl at 0 (control), 6, and 12 hours (**A**). Equality of total RNA concentration was confirmed by 18s rRNA gene expression as an internal control. PCR products were analyzed on 1.2% (w/v) gel electrophoresis precast with SYBR^®^ safe. Relative expression analysis of stressed cells was quantitated using the Image Lab program (**B**). Data are expressed as mean ± xx. * *p* < 0.05, ** *p* < 0.01, *** *p* < 0.001, two-way ANOVA (*n* = 3).

Mechanisms behind salt stress were further investigated by transcriptional analysis. *C. reinhardtii* (137c) was treated with 200 mM NaCl for 0 (control), 6, and 12 hours. Transcriptional expression of genes involved in FA and TAG biosynthesis was examined using RT-PCR. These genes included *PDH2, ACCase, MAT, KAS2, FAT1, GPD1, DGTT1, DGTT2, DGTT3,* and *DGTT4*. Among these genes, *PDH2, ACCase, MAT, KAS2* and *FAT* were involved in the FA biosynthetic pathway, while *GPD1*, and *DGTT1-4* were involved in the Kennedy pathway (TAG biosynthetic pathway). Note that *DGTT* is an alternative name for *DGAT* in *C. reinhardtii* [26]. Figure 3 illustrates gene expression analysis with NaCl stress for 0, 6, and 12 hours. The equality of total RNA concentration was confirmed using 18s *rRNA* as an internal control (intensity was quantitated as 1.0 in all conditions). RT-PCR analysis revealed that the genes were differentially expressed with NaCl stress. The expression of *PDH2*, *MAT* and *KAS2* were significantly up-regulated at 12 hours of salt stress. Expression of *PDH2* and *MAT* was highest at 1.83 ± 0.20 and 1.82 ± 0.06 fold, respectively. *KAS2* expression was significantly increased to 1.70 ± 0.05 fold. *ACCase* was significantly up-regulated (1.87 ± 0.05 fold) after six hours of stress, and then declined slightly. Three genes involved in the Kennedy pathway were down-regulated. *DGTT1* and *DGTT2* expressions were sharply decreased. *FAT1* expression was extremely low, and found to be down-regulated by salt stress (data not shown). Expression of *DGTT3* and *DGTT4* could not be analyzed because of non-specific PCR products (data not shown). Based on gene expression analysis, NaCl triggered the expression of genes involved in the FA biosynthesis pathway. Conversely, NaCl down-regulated genes involved in the Kennedy pathway. It was unexpected that the expression of genes related to TAG synthesis would be decreased by this stress condition, but this observation was consistent with the transient reduction of intracellular lipid content under 200 mM NaCl stress (Figure 1A). Although the microalgal regulatory relationship between intracellular lipid accumulation and biosynthetic pathways under salt stress is not completely understood, several reports have begun to elucidate it. In addition to lipid biosynthesis, salt stress affects starch biosynthesis. In *C. protothecoides*, NaCl treatment decreased the activity of phosphoenolpyruvate carboxykinase and isocitrate dehydrogenase, which play roles in gluconeogenesis and Kreb’s cycle [27]. Therefore, this condition is thought to inhibit glucose synthesis. Furthermore, the activities of enzymes involved in the starch synthesis pathway were down-regulated under salt stress conditions. This supported the hypothesis that salt stress induces a starch-to-lipid shift [12]. 

### 2.4. Generation of Expressing Cyanobacterium Cells Carrying ChMAT and SynMAT

In photosynthetic organisms, such as microalgae, the de novo pathway for lipid biosynthesis is comprised of two main steps: (1) FA biosynthesis that takes place in chloroplasts, and (2) TAG biosynthesis that takes place in the cytosol [28,29,30]. Among enzymes associated with FA synthesis, MAT is not a component of an enzymatic complex, as is the case for PDH and ACCase. In addition, MAT is the enzyme responsible for the production of malonyl ACP, a key building block for FA biosynthesis. As shown in Figure 3, *Chlamydomonas* MAT was up-regulated under salt stress, under the conditions that triggered lipid droplet accumulation, and wherein SFA increase was the most pronounced. We therefore tested whether this gene increased FA content in freshwater cyanobacterium model, *S. elongatus* PCC 7942. In parallel, SynMAT, the MAT gene in *S. elongatus* PCC 7942, was also used for expression in order to compare the results.

In this study, pSyn_6 was used as the expression vector. Thus, expression of the target gene is driven under the strong constitutive promoter *psbA*. The construct polyhistidine tag allows the observation of protein expression by Western blotting. Screening transformed cells was conducted in BG11 supplemented with spectinomycin. Positive clones were re-streaked to obtain an F2 generation. We analyzed 10 independent candidate transformants by colony PCR (data not shown). Our results revealed independent clones for ChMAT and SynMAT containing target genes. Morphological analysis showed no difference in phenotype among wild type, empty vector, ChMAT/7942, and SynMAT/7942, expressing cells cultivated in non-stress conditions (data not shown). 

### 2.5. MAT Transcription was Up-Regulated Depending on Growth Phase in Expressing Cells

**Figure 4:**
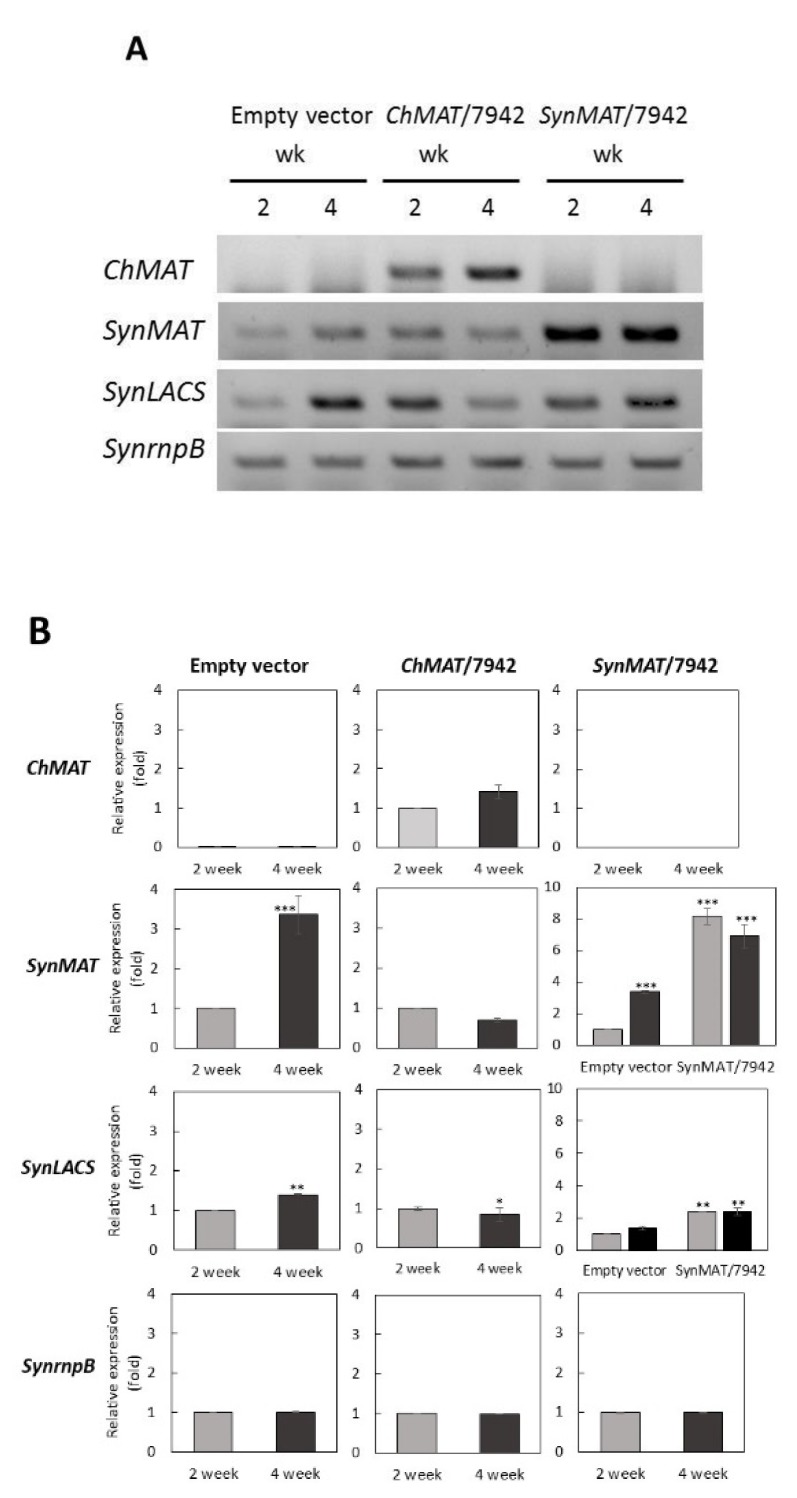
Semi quantitative RT-PCR analysis of cells expressing *ChMAT* and *SynMAT* (**A**). Equality of total RNA concentrations was confirmed by *SynrnpB* gene expression as an internal control. PCR products were analyzed by 1.2% (w/v) gel electrophoresis precast with SYBR^®^ safe. Relative expression analysis of *SynMAT* and *SynLACS* genes were quantitated using the Image Lab program (**B**). * *p* < 0.05, ** *p* < 0.01, paired student t-test (*n* = 3).

Clones harboring empty vector, ChMAT/7942, and SynMAT/7942 were cultured in BG11 supplemented with spectinomycin for two and four weeks, which are the exponential and stationary growth phases, respectively. Equal amount of cDNA template was confirmed by using *RNase P* gene, *SynrnpB*, as an internal control gene (Figure 4). We analyzed the expression of introduced genes (*ChMAT* or *SynMAT*) and *SynLACS* (encoding long-chain acyl-CoA synthetase), the rate-limiting enzyme for FA recycling. 

In the empty vector control, *SynMAT* and *SynLACS* were significantly up-regulated during the stationary growth phase, with a 1.39 ± 0.02 fold increase for *SynMAT* and 3.37 ± 0.48 fold increase for *SynLACS* compared to the exponential phase (Figure 4A,B). This suggested that FA biosynthesis and recycling were both triggered. Up-regulation of these genes would be important for FA recycling and utilization for other components. Expression of the *ChMAT* gene was clearly observed in ChMAT/7942-expressing cells, while *ChMAT* expression was absent in empty vector- and SynMAT/7942-expressing cells (Figure 4A,B). Expression of *ChMAT* was increased 1.41 ± 0.17 fold at the stationary phase. By contrast, in empty vector control cells, both *SynMAT* and *SynLACS* expression were down-regulated in cells expressing ChMAT. In SynMAT-expressing cells, the relative abundance of *SynMAT* increased 8.12 ± 0.10 and 6.89 ± 0.74 fold compared to empty vector controls at exponential and stationary growth phases, respectively. *SynLACS* expression was not statistically changed. 

**Figure 5:**
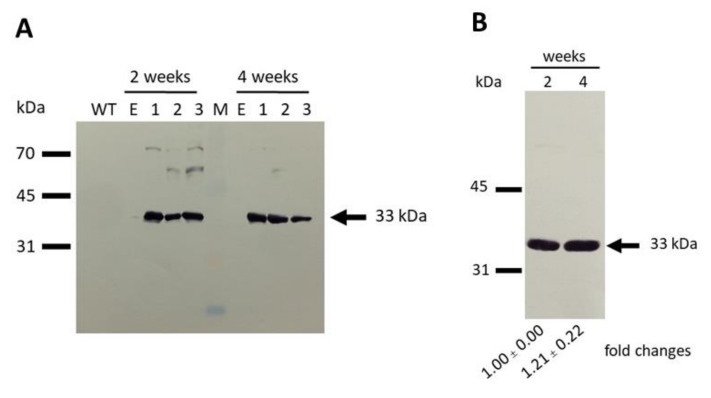
Western blot analysis of empty vector and SynMAT expressing cells. (**A**) wild type, empty vector and SynMAT (2 and 4 weeks old). (**B**) SynMAT (2 and 4 weeks old). An equal amount of crude extract (20 µg) was loaded in all lanes. Blotting was conducted with a PVDF membrane. Antibody raised against 6x His-tag and HRP-conjugated anti-mouse IgG were used as primary and secondary antibodies, respectively. The membranes were developed using a using Horseradish Peroxidase Conjugate Substrate kit.

Western blotting was conducted to observe protein levels in empty vector control cells and clones harboring empty vector, ChMAT/7942, and SynMAT/7942. Because the target gene (s) contained a polyhistidine tag, this facilitates observation of protein expression by Western blotting. We could not detect a specific band in empty vector control constructs (data not shown). Surprisingly, crude extracts prepared from three independent clones harboring ChMAT growing either at exponential or stationary phases did not exhibit a specific band in Western blotting (Figure S5). In contrast, crude extracts prepared from clones harboring SynMAT displayed a specific band of the target fusion protein-His6 at approximately 33 kDa, which was in agreement with the theoretical molecular mass (30.5 kDa) (Figure 5A). These results indicate that ChMAT introduced into *S. elongatus* PCC 7942 was transcribed but not translated. Western blotting revealed a 1.2-fold increase of SynMAT when the expressing cells were in stationary phase (Figure 5B).

### 2.6. Lipid Analysis in Transformants

Two independent transformant cells expressing ChMAT/7942 and SynMAT/7942 were collected at the exponential growth phase (OD730 ~ 0.8–1.0). The cells were extracted and FAs were analyzed by FAMEs, as described in Materials and Methods. Table 1 demonstrates that the expression of heterologous ChMAT and empty vector control produced similar cellular FA profiles and compositions. However, the total FA of SynMAT transformants significantly increased (Table 1). 

However, heterologous expression of ChMAT was observed at the transcriptional level, but not translational level (Figure 4 and Figure 5). Different codon usage between *C. reinhardtii* (137c) and *S. elongatus* PCC 7942 might affect the translation step, as codon usage is known to be genome specific and related to taxonomic order [31]. Thus, the constant lipid content and profile in ChMAT/7942 may be altered because protein accumulation appeared to be absent or extremely low. In SynMAT/7942, the transcriptional level was highly up-regulated. The translational level was also considerably higher (Figure 5B). Table 1 shows that the overexpression of SynMAT increased the levels of palmitic acid (C16:0) and stearic acid (C18:0). A slight decrease in palmitoleic acid (C16:1) and oleic acid (C18:1) was also observed. These results suggest that salt stress impacts only C16 and C18 synthesis.

Recently, overexpression of putative *fabD* (*SynMAT*) under the regulation of the *trc* promoter in *S. elongatus PCC 7942* was found to alter lipid composition. Increases of myristic acid (C14:0) and palmitoleic acid (C16:1) were detected, while the levels of palmitic acid (C16:0), stearic acid (C18:0), and oleic acid (C18:1) were decreased [32]. Although the same gene was used in this study as in a report by Santos-Merino et al. [32], the promoter used in our study is the *psbA* promoter, which is constitutively expressed. This could potentially explain these differential findings. In *E. coli*, overexpression of MAT under regulation of the *trc* promoter significantly increased total FAs. Myristic acid (C14:0) and palmitoleic acid (C16:1) were increased, while palmitic acid (C16:0) was unchanged [33]. However, when MAT was expressed under the lac promoter in *E. coli*, oleic acid (C18:1) was increased, while palmitoleic acid (C16:1) was decreased [34]. This suggested that different promoters had differential effects in the cellular lipid profile.

## 3. Materials and Methods

### 3.1. Strains and Growth Conditions

*C. reinhardtii* (137c) cells were grown photoautotrophically (50 μmol m^−2^s^−1^) in Tris-Acetate-Phosphate (TAP) medium at 28 ± 2 °C (Invitrogen, Carlsbad, CA, USA). *S. elongatus* PCC 7942 cells were grown photoautotrophically (50 μmol m^−2^s^−1^) in blue-green 11 (BG11) liquid medium at 28 ± 2 °C. *Escherichia coli* strain DH5α cells were grown in Luria-Bertani (LB) medium at 37 °C. The growth of *E. coli* or microalgal cells was monitored by measuring the absorbance at 600 nm or 730 nm, respectively, using a UV-240 spectrophotometer (Shimadzu, Kyoto, Japan).

### 3.2. Stress Treatments

Prior to the salt stress experiments, microalgal cells were grown photoautotrophically in a growth medium until it reached the exponential phase. For the salt shock experiments, cells were harvested by centrifugation at 12,000 rpm at 4 °C for 10 minutes. Cell pellets were washed twice with fresh TAP or BG11 media before being subjected to new media containing salts (NaCl, KCl, or LiCl) at the indicated concentrations. Salt stress was induced for 0, 3, 5, and 7 days. Thereafter, the stressed cells were collected and used for lipid analysis.

### 3.3. Lipid Analysis

Nile red (9-diethylamino-5H-benzo[α]phenoxazine-5-one) was used as a lipophilic stain for intracellular TAG detection. Control and stressed cells were harvested by centrifugation at 12,000 rpm for 10 minutes. The supernatant was discarded, and cell pellets were stained with Nile red solution (0.25 mg/mL dissolved in acetone) and 15% DMSO at a ratio of 1:5 (v/v). The mixture was dropped onto a glass slide and covered with a covered slip. Lipid droplets in cells were observed using a fluorescent microscope (Olympus model BX51, Tokyo, Japan). Images were captured using DPController microscope software. Relative intensity of lipid droplets was quantified using ImageJ software (https://imagej.nih.gov/ij/). Statistical significance was analyzed by GraphPad Prism^®^ program (version 5, Graphpad, San Diego, CA, USA) (https://www.graphpad.com/). 

The intracellular FA profile was assessed with FA methyl ester (FAME) analysis. Cell pellets were harvested after control and stress treatments, and were dehydrated using a freeze dryer (Flexi-Dry MP, Kinetics, Boston, MA, USA). Intracellular FAs were extracted from the cell powder with a methanol-hydrochloric acid solution (95:5 V/V). The resulting solution was mixed thoroughly and incubated at 85 °C for 90 min. Distilled water (1 mL) was then added to the mixture. FAME extraction was conducted at room temperature with hexanes containing 0.01% butylated hydroxytoluene. The extracts were centrifuged and separated into an organic phase and an aqueous phase. Sodium sulfate was added to the organic phase to remove water. Thereafter, the FAMEs were evaporated and dry residues were redissolved in hexanes. Subsequently, the extracts were injected into a HP-INNOWax column (ID 0.25 µm) and analyzed with a gas chromatography system (Agilent 6890N, Agilent, Wilmington, DE, USA) equipped with a flame ionization detector (FID). The injection and detection temperatures were maintained at 250 °C. Helium gas was used as the mobile phase. Extracted FA content was calculated on comparison with 14 authentic compounds (caprylic acid, capric acid, lauric acid, myristic acid, palmitic acid, palmitoleic acid, stearic acid, oleic acid, linoleic acid, linolenic acid, arachidonic acid, behenic acid, erucic acid, and lignoceric acid).

### 3.4. Gene Expression Analysis

Total RNA was extracted from control and stressed cells using TRIzol^^^®^^^ reagent (Invitrogen, USA) according to the manufacturer’s instructions. RNA concentration and quality were determined using a Nanodrop 2000 (Thermo Scientific, Waltham, MA, USA) and gel electrophoresis, respectively. For cDNA synthesis, 3000 ng of total RNA was reverse transcribed using the SuperScript^®^III First-Stranded synthesis kit (Invitrogen, CA) according to the manufacturer’s instructions. Semi-quantitative RT-PCR analysis was conducted to examine transcriptional levels of genes involved in lipid biosynthesis. PCR amplification was performed with specific primer pairs (Table S2). The 18s rRNA gene was used as an internal control. The PCR products were electrophoresed on a 1.2% (w/v) agarose gel. Relative intensity was quantified using Image Lab^TM^ software (version 6.0, Bio-Rad, Hercules, CA, USA) (http://www.bio-rad.com/en-th/product/image-lab-software?ID=KRE6P5E8Z), and statistical difference was assessed with a two-way ANOVA using the GraphPad Prism^®^ program, Version 5.

### 3.5. Construction of Expressing Cells

The encoding region of *ChMAT* (the gene encoding MAT from *C. reinhardtii* (137c); accession number XP_001689862) was amplified by polymerase chain reaction (PCR) from *C. reinhardtii* (137c) cDNA using specific primer pairs, including ChMAT_NdeI_Forward and ChMAT_BamHI_Reverse (Table S2), which contain restriction target NdeI and BamHI sites and are cloned into the pBluescript II SK(+) vector (Toyobo, Osaka, Japan) and sequenced. Thereafter, *ChMAT* was transferred into the expression vector pSyn_6 (Invitrogen, USA) at corresponding restriction sites, generating pSyn_6_ChMAT. The encoding region of *SynMAT* (the gene encoding MAT from *S. elongatus* PCC 7942; accession number ABB57486) was amplified by PCR from *S.elongatus* PCC 7942 genomic DNA using the specific primer pairs SynMAT_NdeI_Forward and SynMAT_BamHI_Reverse (Table S2). Cloning and introduction of SynMAT into pBluescript II SK(+) and pSyn_6 vectors were performed using the same protocol described for *ChMAT*, generating pBluescript II SK_SynMAT and pSyn6_SynMAT, respectively.

Expressing vectors harboring target genes, including pSyn_6_ChMAT and pSyn_6_SynMAT were transformed into *S. elongatus* PCC 7942 as previously described [35]. Candidate transformants were selected on BG11 agar supplemented with spectinomycin (10 μg/mL). Independent transformants were verified, and the presence of target genes was confirmed by colony PCR.

### 3.6. Other Methods

SDS-PAGE and Western blot analyses were performed according to standard protocols. All molecular cloning methods were performed according to established procedures [36]. Protein concentrations were determined by the Bradford method using bovine serum albumin as the standard protein. Anti-6-His (6-His tag) (R&D Systems, Minneapolis, MN, USA) and anti-mouse IgG-HRP conjugated (New England Biolab, Ipswich, MA, USA) were used as primary and secondary antibodies, respectively. Nucleotide sequences were determined by Eurofins Genomics, Tokyo, Japan.

## 4. Conclusions

In summary, the effects of salt-induced stresses on lipid and FA compositions in green microalga were elucidated. NaCl-induced stress dramatically increased SFA, but decreased PUFA composition. Unlike NaCl stress, a simple stress by KCl slightly modulated SFA and PUFA compositions. Expression analysis revealed that the genes involved in FA biosynthesis were up-regulated under salt stress. SynMAT overexpression in a fresh water cyanobacterium altered FA composition and elevated lipid content that might be employed to generate biofuel with improved quality. As recent decades have been marked by an explosive growth of interest in the use of microalgae lipid as a source of valuable nutritional ingredients, in biotechnological industries, and as precursors for biodiesel production, our findings demonstrate a remarkable solution toward increase in the overall yield or shift in the balance of algal lipid metabolism for microalgal lipid production.

## Figures and Tables

**Table 1:** Lipid profile of ChMAT/7942 and SynMAT/7942 cells at the exponential growth phase. ChMAT/7942 and SynMAT/7942 cells were collected and lipids were extracted, as described in Materials and Methods. (ND; not detected). Significant differences between the mean values of control and stress treatment were assessed by a two-tailed Student’s t-test. * *p* < 0.01, ** *p* < 0.001, *** *p* < 0.0001 (*n* = 3).

Fatty Acid (%) (Methyl Ester)	Empty Vector	ChMAT/7942	SynMAT/7942
SATURATED FATTY ACIDS (SFAs)			
Caprylic acid (C8:0)	0.07	0.10	0.07
Capric acid (C10:0)	0.00	0.00	0.00
Lauric acid (C12:0)	0.00	0.00	0.00
Capric acid (C10:0)	0.00	0.00	2.09
Lauric acid (C12:0)	0.00	0.00	48.74 ***
Myristic acid (C14:0)	2.07	2.12	1.12*
Palmitic acid (C16:0)	42.93	43.52	0.00
Stearic acid (C18:0)	0.36	0.30	0.00
Arachinodic acid (C20:0)	0.00	0.00	0.00
Behenic acid (C22:0)	0.00	0.00	52.00 ***
MONO-UNSATURATED FATTY ACIDS (MUFAs)			
Palmitoleic acid (C16:1)	49.38	49.42	43.91 ***
Oleic acid (C18:1)	4.99	4.41	3.80 ***
Erucic acid (C22:1)	0.00	0.00	0.00
Total MUFA	C_58_H_82_O_5_	53.83	47.71 ***
POLY-UNSATURATED FATTY ACIDS (PUFAs)			
Linoleic acid (C18:2)	0.20	0.20	0.29
Linolenic acid (C18:3)	0.00	0.00	0.00
Total PUFA	0.20	0.20	0.29

## Supplementary Materials

The following are available online at https://www.mdpi.com/1660-3397/17/8/484/s1, Figure S1: *C. reinhardtii* (137c) cells observed under a bright field light microscope (BF) and fluorescent microscope (NR). Cells at the exponential growth phase were subjected to salt stress (NaCl) at various concentrations, including 0 mM, 50 mM, 100 mM, 150 mM, 200 mM, and 250 mM for 0, 3, 5, and 7 days. Morphology and lipid bodies were observed by Nile red staining; Figure S2: *C. reinhardtii* (137c) cells observed under a bright field light microscope (BF) and fluorescent microscope (NR). Cells at the exponential growth phase were subjected to salt stress (KCl) at various concentrations, including 0 mM, 50 mM, 100 mM, 150 mM, 200 mM, and 250 mM for 0, 3, 5, and 7 days. Morphology and lipid bodies were observed by Nile red staining; Figure S3: *C. reinhardtii* (137c) cells observed under a bright field light microscope (BF) and fluorescent microscope (NR). Cells at the exponential growth phase were subjected to salt stress (LiCl) at various concentrations, including 0 mM, 30 mM, 60 mM, 90 mM, 120 mM, and 150 mM for 0, 3, 5, and 7 days. Morphology and lipid bodies were observed by Nile red staining; Figure S4: *C. reinhardtii* (137c) cells observed under a light microscope (A-F) and fluorescent microscope (G-L). Cells at the exponential growth phase were subjected to sodium acetate at various concentrations, including 0 mM (A, G), 50 mM (B, H), 100 mM (C, I), 150 mM (D, J), 200 mM (E, K), and 250 mM (F, L) for 7 days. Morphology and lipid droplets were observed by Nile red staining as described in Materials and Methods. Relative intensity of lipid content in algal cells under sodium acetate stress was analyzed using ImageJ software (M). Data are expressed as mean ± SE. * *p* < 0.01, ** *p* < 0.001, unpaired T-test (n ≥ 10); Table S1: Intracellular fatty acid composition of *C. reinhardtii* (137c) cells subjected to salt stresses (NaCl, KCl, and LiCl) at days 3, 5, and 7. One-hundred milligrams DCW of each stressed cell were collected, and lipids were extracted as described in Materials and Methods. Data are expressed as mean ± SD. ND: not detected. Significant differences between the mean values of control and stress treatments were assessed by a two-tailed Student’s t-test. * *p* < 0.01, ** *p* < 0.001 and *** *p* < 0.0001 (*n* = 3); Table S2: primers used in this study.
