# Supplementary material for: Enhanced Lipid Production and Molecular Dynamics under Salinity Stress in Green Microalga Chlamydomonas reinhardtii (137C)
Source: Mar Drugs. 2019 Aug 20;17(8):484. doi: 10.3390/md17080484 (PMC6722929; doi:10.3390/md17080484)
Supplement: Supplementary file 1 [file marinedrugs-17-00484-s001.pdf]

Table S1 Intracellular fatty acid composition of *C. reinhardtii* (137c) cells subjected to salt stresses (NaCl, KCl, and LiCl) at days 3, 5, and 7. One-hundred milligrams DCW of each stressed cell were collected, and lipids were extracted as described in the Materials and Methods. Data are expressed as mean ± SD. ND: not detected. Significant differences between the mean values of control and stress treatments were assessed by a two-tailed Student's t-test. *$p < 0.01$, **$p < 0.001$ and ***$p < 0.0001$ (n = 3).

Fatty acid (%)	Control	200 mM NaCl			200 mM KCl			120 mM LiCl		
		3 days	5 days	7 days	3 days	5 days	7 days	3 days	5 days	7 days
**SATURATED FATTY ACIDS (SFAs)**										
Caprylic acid (C8:0)	ND	ND	ND	ND	ND	ND	ND	ND	ND	ND
Capric acid (C10:0)	ND	ND	ND	ND	ND	ND	ND	ND	ND	ND
Lauric acid (C12:0)	ND	0.02 ± 0.00	0.01 ± 0.02	0.03 ± 0.03	ND	0.01 ± 0.00	ND	0.05 ± 0.00	0.05 ± 0.00	0.03 ± 0.00
Myristic acid (C14:0)	1.93 ± 0.03	0.57 ± 0.08	1.78 ± 1.49	2.24 ± 1.97	2.80 ± 0.10	2.98 ± 0.06	3.28 ± 0.13	0.66 ± 0.01	0.67 ± 0.01	0.07 ± 0.01
Palmitic acid (C16:0)	31.94 ± 0.16	43.14 ± 10.56	51.20 ± 14.96	59.80 ± 14.46	36.20 ± 0.00	36.28 ± 0.01	39.32 ± 0.03	35.98 ± 0.04	36.57 ± 0.03	37.51 ± 0.03
Stearic acid (C18:0)	4.70 ± 0.04	6.47 ± 2.68	6.78 ± 3.85	7.75 ± 4.69	3.70 ± 0.03	3.80 ± 0.03	4.16 ± 0.02	4.30 ± 0.04	4.39 ± 0.00	4.64 ± 0.00
Arachinodic acid (C20:0)	0.22 ± 0.00	0.28 ± 0.06	0.27 ±0.13	0.37 ± 0.13	0.22 ± 0.00	0.26 ±0.01	0.29 ± 0.00	0.21 ± 0.01	0.22 ±0.00	0.21 ± 0.00
Behenic acid (C22:0)	ND	ND	ND	ND	ND	ND	ND	ND	ND	ND
Lignoceric acid (C24:0)	ND	ND	ND	ND	ND	ND	ND	ND	ND	ND
**Total SFA**	**38.79 ± 0.33**	**50.48 ± 6.67*****	**60.04 ± 5.66*****	**70.19 ± 0.00*****	**42.92 ± 0.01*****	**43.33 ± 2.85*****	**47.05 ± 0.00*****	**41.20 ± 0.38****	**41.90 ± 0.22*****	**43.09 ± 0.00*****
**MONO-UNSATURATED FATTY ACIDS (MUFAs)**										
Palmitoleic acid (C16:1)	0.22 ± 0.25	0.47 ± 0.38	0.55 ± 0.41	0.56 ± 0.39	0.40 ± 0.37	0.48 ± 0.36	0.53 ± 0.54	0.60 ± 0.31	0.56 ± 0.31	0.50 ± 0.06
Oleic acid (C18:1)	5.69 ± 1.70	7.20 ± 3.48	6.20 ± 2.84	5.22 ± 2.80	5.05 ± 0.99	5.91 ± 0.18	6.55 ± 3.52	3.09 ± 2.65	3.38 ± 2.97	1.58 ± 0.68
Erucic acid (C22:1)	ND	ND	ND	ND	ND	ND	ND	ND	ND	ND
**Total MUFA**	**5.91 ± 0.66**	**7.67 ± 0.00**	**6.75 ± 0.00**	**5.78 ± 0.00**	**5.45 ± 1.29**	**6.39 ± 0.16**	**7.08 ± 0.00**	**3.69 ± 0.00***	**3.94 ± 0.00**	**2.08 ± 0.00*****
**POLY-UNSATURATED FATTY ACIDS (PUFAs)**										
Linoleic acid (C18:2)	33.64 ± 0.04	26.65 ± 7.37	22.14 ± 8.52	15.93 ± 12.23	29.51 ± 2.35	30.16 ± 5.54	28.48 ± 18.94	34.19 ± 2.73	34.42 ± 3.59	36.97 ± 7.98
Linolenic acid (C18:3)	22.94 ± 2.36	13.60 ± 6.60	11.95 ± 7.37	7.13 ± 6.42	22.20 ± 1.61	18.22 ± 2.19	17.29 ± 11.81	20.96 ± 6.10	19.24 ± 5.54	17.75 ± 8.74
**Total PUFA**	**56.58 ± 1.03**	**40.25 ± 0.03*****	**34.09 ± 2.11*****	**23.06 ± 0.00*****	**51.71 ± 0.74*****	**44.47 ± 1.55*****	**45.77 ± 0.00*****	**55.15 ± 2.73**	**53.66 ± 3.59*****	**54.72 ± 0.00**

Table S2 Primer used in this study.

Primers	Sequences (5' - 3')	Base pairs
**ChlmyPDH2_Forward**	CCCATTGTGGAGGGC	15
**ChlmyPDH2_Reverse**	ACAGCAGAACGTGCT	15
**ChlamyACCase_Forward**	TCTGCAAGTCGCTCG	15
**ChlamyACCase_Reverse**	CGTGTCCACGAAGGT	15
**ChlamyMAT_Forward**	CCGTGAGTCAGCCCG	15
**ChlamyMAT_Reverse**	TGGACCGGCTTGTCC	15
**ChlamyKAS2_Forward**	CGCGGACTACACGAC	15
**ChlamyKAS2_Reverse**	GGCGAAGGGAATGCA	15
**ChlamyGPD1_Forward**	CAAAGGCCTTGAGGT	15
**ChlamyGPD1_Reverse**	CAAGTCCATGCCCTC	15
**ChlamyFAT 1_Forward**	TCAGGAGTCGCGGTG	15
**ChlamyFAT 1_Reverse**	GCACTTGCAGGCGTG	15
**ChlamyDGTT1_Forward**	GATTAAGACCGCCGA	15
**ChlamyDGTT1_Reverse**	AGCGCCTCAGACGCG	15
**ChlamyDGTT2_Forward**	TCCTGTCCTTCTGGT	15
**ChlamyDGTT2_Reverse**	TGAACACCACATTCG	15
**ChlamyDGTT3_Forward**	TACCTCGCACTTGAC	15
**ChlamyDGTT3_Reverse**	AAGTAGTGACGCCAG	15
**ChlamyDGTT4_Forward**	GGCTACTTGTTCGGA	15
**ChlamyDGTT4_Reverse**	GCTGGAGAGGTAGGC	15
**Chlamy18s rRNA_Forward**	CGGAACCCGCTGGTC	15
**Chlamy18s rRNA_Reverse**	TGAAGGTGAGCGGCG	15
**ChMAT_NdeI_Forward**	ATCATATGGTCGCTGTCC	18
**ChMAT_BamHI_Reverse**	GGATCCAGCGGTGAT	16
**SynMAT_NdeI_Forward**	CATATGGCTAAAACGGTGTGGGTGT	25
**SynMAT_BamHI_Reverse**	GGATCCGACCGTGAGGTCTGC	21
**pSyn_psbA**	TCTTGCCCTTTACAACCT	18
**pSyn_rrnB**	CGCTACTGCCGCCAG	15
**7942rnpB_Forward**	GAGGAAAGTCCGGGCTCCC	19
**7942rnpB _Reverse**	TAAGCCGGGTTCTGTTCTC	19
**7942MAT_Forward**	CAGCCCTCTACACCG	15
**7942MAT_Reverse**	GCCATAAACGGCGAG	15
**7942LACS_Forward**	TGCTTTCTCACGGCA	15
**7942LACS_Reverse**	TCGATCAAGCGACGT	15

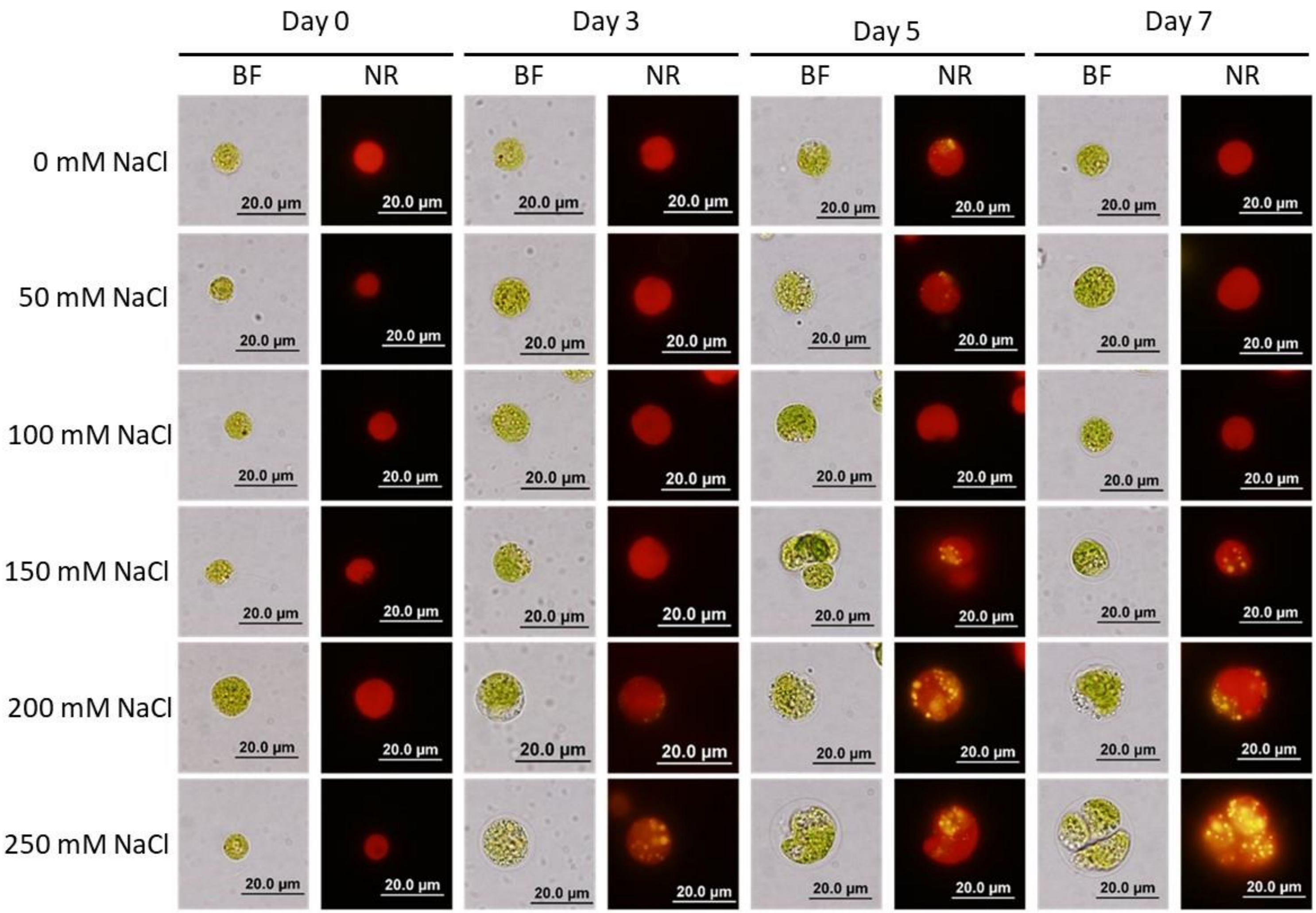

Figure S1 *C. reinhardtii* (137c) cells observed under a bright field light microscope (BF) and fluorescent microscope (NR). Cells at the exponential growth phase were subjected to salt stress (NaCl) at various concentrations, including 0 mM, 50 mM, 100 mM, 150 mM, 200 mM, and 250 mM for 0, 3, 5, and 7 days. Morphology and lipid bodies were observed by Nile red staining.

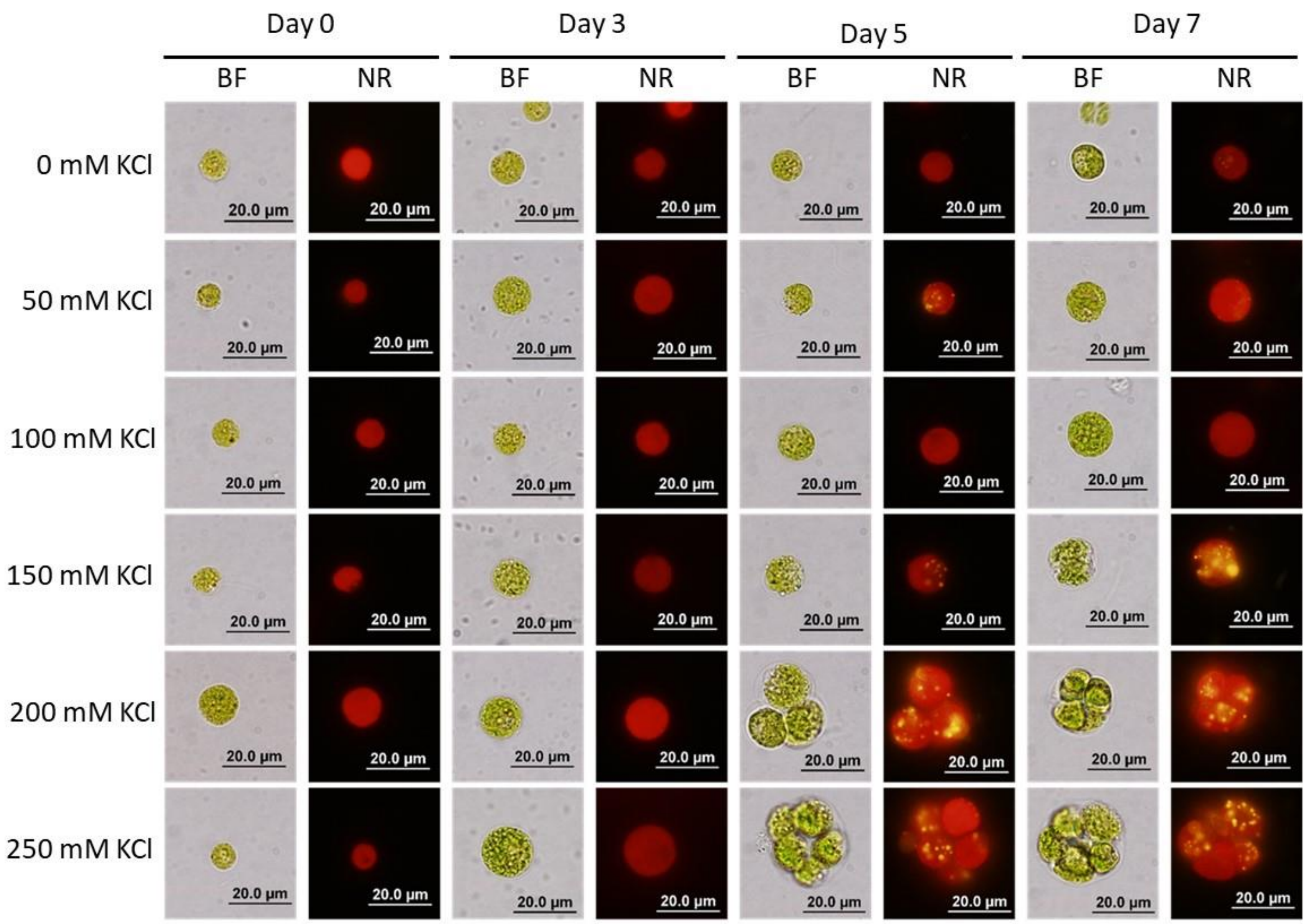

Figure S2 *C. reinhardtii* (137c) cells observed under bright field light microscope (BF) and fluorescent microscope (NR). Cells at exponential growth phase were subjected to salt stress (KCl) at various concentrations; 0 mM, 50 mM, 100 mM, 150 mM, 200 mM, and 250 mM for 0, 3, 5, and 7 days. Morphology and lipid body were observed by Nile red staining.

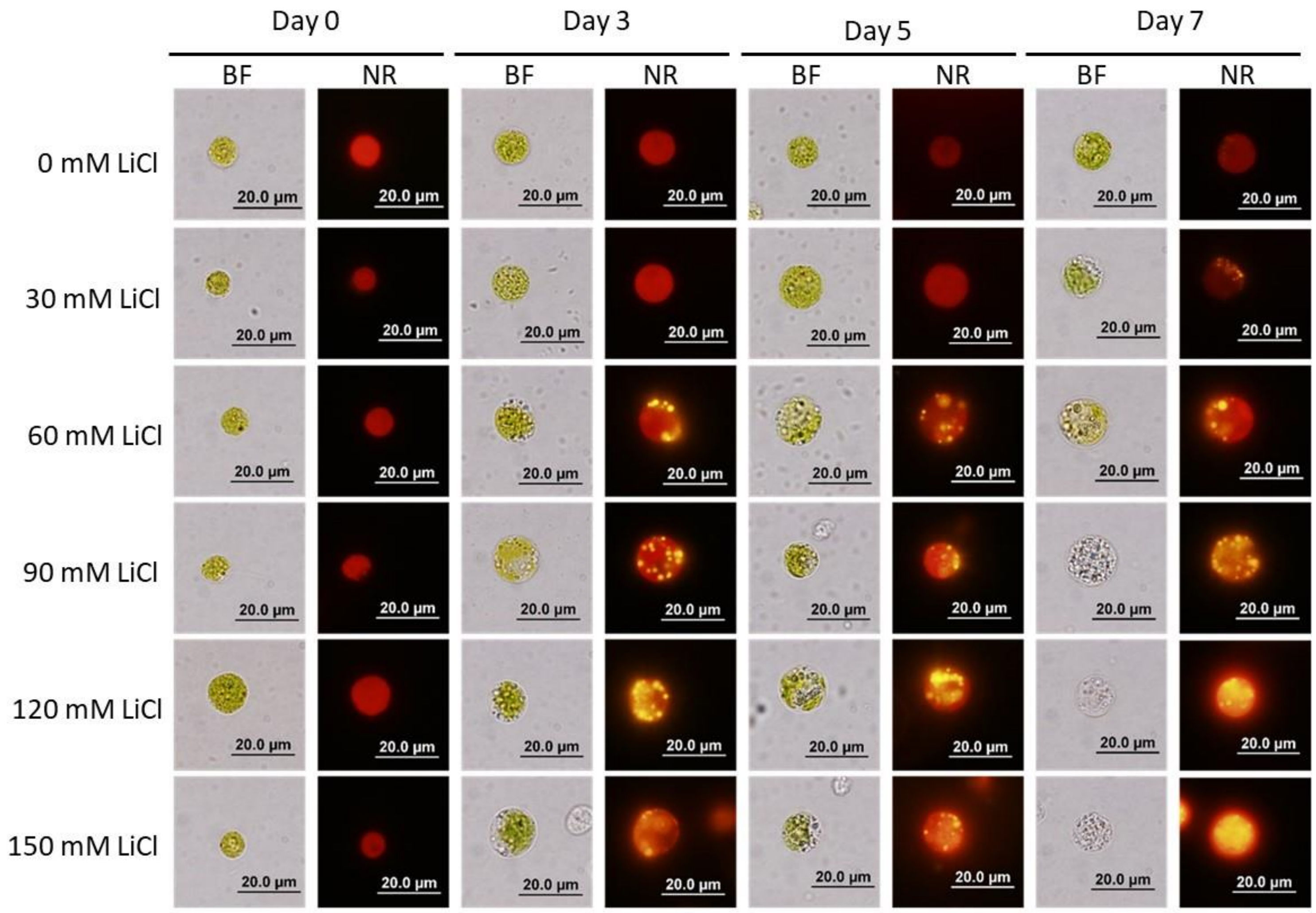

Figure S3 *C. reinhardtii* (137c) cells observed under a bright field light microscope (BF) and fluorescent microscope (NR). Cells at the exponential growth phase were subjected to salt stress (LiCl) at various concentrations, including 0 mM, 30 mM, 60 mM, 90 mM, 120 mM, and 150 mM for 0, 3, 5, and 7 days. Morphology and lipid bodies were observed by Nile red staining.

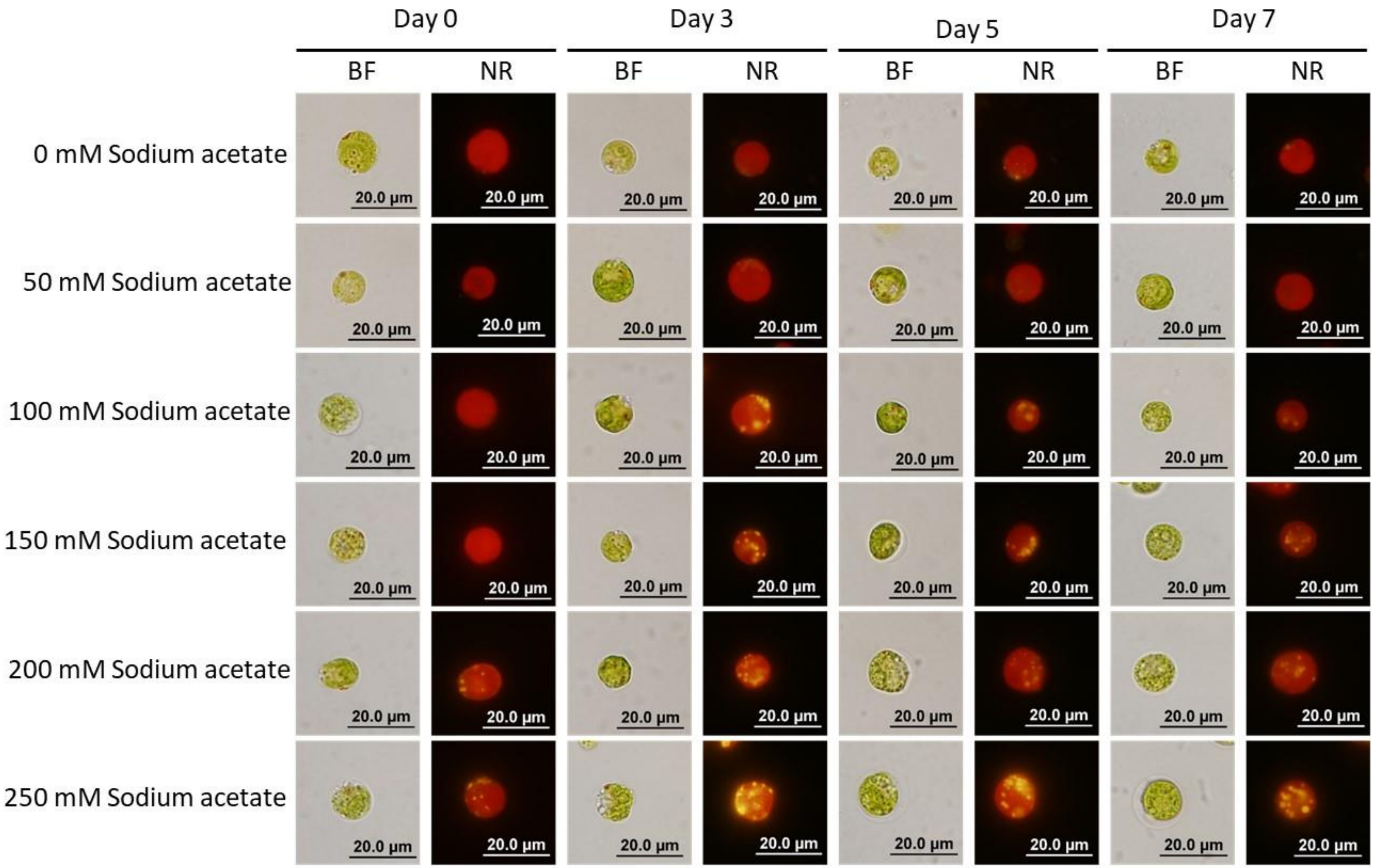

Figure S4 *C. reinhardtii* (137c) cells observed under a bright field light microscope (BF) and fluorescent microscope (NR). Cells at the exponential growth phase were subjected to sodium acetate at various concentrations, including 0 mM, 50 mM, 100 mM, 150 mM, 200 mM, and 250 mM for 0, 3, 5, and 7 days. Morphology and lipid bodies were observed by Nile red staining.

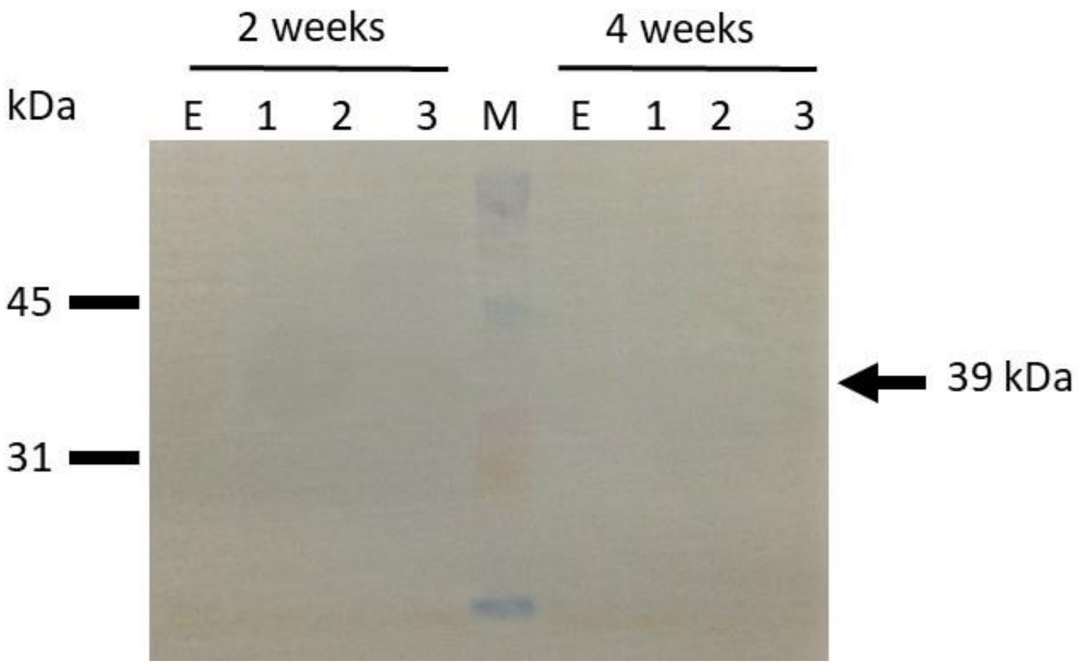

Figure S5 Western blot analysis of empty vector and ChMAT expressing cells. Empty vector (E) and ChMAT (2 and 4 weeks old). An equal amount of crude extract (20 µg) was loaded in all lanes. Blotting was conducted with a PVDF membrane. Antibody raised against 6x His-tag and HRP-conjugated anti-mouse IgG were used as primary and secondary antibodies, respectively. The membranes were developed using a using Horseradish Peroxidase Conjugate Substrate kit.
